# Dysregulation of mitotic machinery genes precedes genome instability during spontaneous pre-malignant transformation of mouse ovarian surface epithelial cells

**DOI:** 10.1186/s12864-016-3068-5

**Published:** 2016-10-25

**Authors:** Ulises Urzúa, Sandra Ampuero, Katherine F. Roby, Garrison A. Owens, David J. Munroe

**Affiliations:** 1 Laboratorio de Genómica Aplicada, Programa de Biología Celular y Molecular, ICBM-Facultad de Medicina, Universidad de Chile, Independencia 1027, Santiago, Chile; 2 Programa de Virología, ICBM-Facultad de Medicina, Universidad de Chile, Independencia 1027, Santiago, Chile; 3 Department of Anatomy & Cell Biology, University of Kansas Medical Center, Kansas City, KS USA; 4 Laboratory of Molecular Technology, NCI-SAIC Frederick, Frederick, MD USA; 5 Cancer Research Technology Program, Leidos Biomedical Research, Inc., Frederick National Laboratory for Cancer Research, Frederick, MD USA; 6 Current address: Life Sciences Solutions Group, ThermoFisher Scientific, 5792 Van Allen Way, Carlsbad, CA 92008 USA

**Keywords:** Ovarian cancer model, Preneoplasia, Mouse ovarian surface epithelium, Transcriptome, Genome, Aneuploidy, Cytokinesis, DNA microarrays

## Abstract

**Background:**

Based in epidemiological evidence, repetitive ovulation has been proposed to play a role in the origin of ovarian cancer by inducing an aberrant wound rupture-repair process of the ovarian surface epithelium (OSE). Accordingly, long term cultures of isolated OSE cells undergo in vitro spontaneous transformation thus developing tumorigenic capacity upon extensive subcultivation. In this work, C57BL/6 mouse OSE (MOSE) cells were cultured up to passage 28 and their RNA and DNA copy number profiles obtained at passages 2, 5, 7, 10, 14, 18, 23, 25 and 28 by means of DNA microarrays. Gene ontology, pathway and network analyses were focused in passages earlier than 20, which is a hallmark of malignancy in this model.

**Results:**

At passage 14, 101 genes were up-regulated in absence of significant DNA copy number changes. Among these, the top-3 enriched functions (>30 fold, *adj p* < 0.05) comprised 7 genes coding for *centralspindlin*, *chromosome passenger* and *minichromosome maintenance* protein complexes. The genes *Ccnb1* (Cyclin B1), *Birc5* (Survivin), *Nusap1* and *Kif23* were the most recurrent in over a dozen GO terms related to the mitotic process. On the other hand, *Pten* plus the large non-coding RNAs *Malat1* and *Neat1* were among the 80 down-regulated genes with *mRNA processing*, *nuclear bodies*, *ER-stress response* and *tumor suppression* as relevant terms. Interestingly, the earliest discrete segmental aneuploidies arose by passage 18 in chromosomes 7, 10, 11, 13, 15, 17 and 19. By passage 23, when MOSE cells express the malignant phenotype, the dysregulated gene expression repertoire expanded, DNA imbalances enlarged in size and covered additional loci.

**Conclusion:**

Prior to early aneuploidies, overexpression of genes coding for the mitotic apparatus in passage-14 pre-malignant MOSE cells indicate an increased proliferation rate suggestive of replicative stress. Concomitant down-regulation of nuclear bodies and RNA processing related genes suggests altered control of nuclear RNA maturation, features recently linked to impaired DNA damage response leading to genome instability. These results, combined with cytogenetic analysis by other authors in this model, suggest that transcriptional profile at passage 14 might induce cytokinesis failure by which tetraploid cells approach a near-tetraploid stage containing primary chromosome aberrations that initiate the tumorigenic drive.

**Electronic supplementary material:**

The online version of this article (doi:10.1186/s12864-016-3068-5) contains supplementary material, which is available to authorized users.

## Background

Non-heritable, sporadic ovarian cancer (OC) continues to be the major cause of death by gynecological cancer in western countries [1]. Early detection markers of OC are not yet available resulting in diagnosis at advanced stages with poor prognosis. Nearly 90 % of OC tumors including carcinomas, cystadenomas, and borderline tumors are histologically related to the ovarian surface epithelium (OSE), a single layer of flat to cuboidal cells wrapping the ovary [2]. Importantly, repeated wound damage-repair of the OSE due to continuous, uninterrupted ovulatory cycles, remains as one of the current mainstream views proposed as OC initiating mechanism [3]. An alternative, recent notion, suggest that OC of the serous carcinoma type originates from the fallopian tube epithelium [4].

The uninterrupted ovulation theory finds additional support in epidemiological data on reproductive history of women. Conditions with reduced number of ovulation cycles such as pregnancies, anovulatory contraception and breastfeeding, confer a significantly reduced OC risk [5–7]. Conversely, continuous ovulation as in nulliparity, increases OC risk [8]. Ovulation is essentially a pro-inflammatory process triggered by a luteinizing hormone (LH) surge leading to increase of reactive oxygen species (ROS) levels in ovarian follicles [9]. Indeed, ROS scavengers placed in the ovarian bursa of mice can impair ovulation [10]. At the site of follicular rupture, OSE cells undergo apoptosis and adjacent cells become exposed to oxidants and inflammatory signals [11].

The OSE is an extension of the peritoneal mesothelium. It is loosely attached to a basement membrane that separates it from the underlying stroma rich in dense collagenous fibers [2]. Since the OSE express epithelial, mesothelial and mesenchymal markers, it has been considered as an “uncommitted” or mixed epithelium. OSE cells additionally express hormone receptors for gonadotropins (FSHR, LHR, GnRH type I), activin/inhibin (ACVR), estrogen (ERα/β) and progesterone (PRA/B) [2]. As the ovary reaches reproductive senescence, OSE cells invaginate to form cortical epithelial crypts and inclusion cysts that have been proposed to be pre-neoplastic lesions [12]. In contrast to the mixed phenotype of the OSE, inclusion cysts predominantly express epithelial markers, a phenotype consistent with an atypical reverse epithelial-mesenchymal transition (EMT) observed in OC tumors [13]. While early ovarian carcinomas display increased expression of E-cadherin, an epithelial cell marker, advanced OC cells partially recover mesenchymal features, thus decreasing E-cadherin expression [14].

Due to its proposed relevance in OC initiation, OSE cells from diverse sources have been characterized as experimental models of ovarian carcinogenesis. Rodent OSE cells detached from the ovaries undergo spontaneous transformation after repeated subcultivation [15, 16]. Furthermore, when injected in the peritoneal cavity of immunocompetent animals, such spontaneously transformed mouse OSE (MOSE) cells induce tumor implants and hemorrhagic ascites [15, 17], features typical of advanced human OC [18]. Therefore, the proliferative pressure imposed on cultured MOSE cells seems to induce a transformation path resembling the naturally in vivo process of repetitive wound damage-repair.

Importantly, the progression from a pre-malignant non-tumorigenic to an aggressive phenotype has been monitored in the MOSE culture model [15, 17, 19–21]. Altered actin cytoskeleton, reduced focal adhesion plaques, E-cadherin down-regulation and subcellular mislocalization of connexin-43 (*Gja1*) among other parameters, have been used to define sequential stages of cell transformation [19]. Massive cell cytoskeleton disorganization was found to be mediated by dysregulation of about 140 genes coding for components of actin filaments, microtubules and intermediate filaments. In addition, altered global patterns of ser/tyr phosphorylation plus APC and PKCII mislocalization paralleled the remodeling of cellular architecture [20].

Cytogenetic changes have been also studied in the MOSE model [21]. By passage 19, diploid and tetraploid cells coexisted. By passage 26, mononucleated tetraploid cells - initially binucleated diploid due to citokinesis failure- become predominant over undetectable diploid cells. Bipolar or multipolar mitosis occasionally occurs during proliferation of mononucleated tetraploid cells leading to chromosome mis-segregation and near-tetraploid aneuploidy [21]. In the present work we aimed to uncover gene networks underlying very early stages of MOSE transformation. At passage 14, we detected dysregulation of three key protein complexes and numerous additional genes involved in chromosome dynamics during cell division. In addition, the first detectable aneuploidies were identified at passage 18. We propose that these early transcriptional alterations would be both primarily implicated in cytokinesis failure of initial diploid cells, and also in anomalous mitosis leading to aneuploidy and tumorigenicity of late-passage, tetraploid MOSE cells.

## Results and discussion

### Strategy for genomic and transcriptional profiling of MOSE cells

**Fig. 1:**
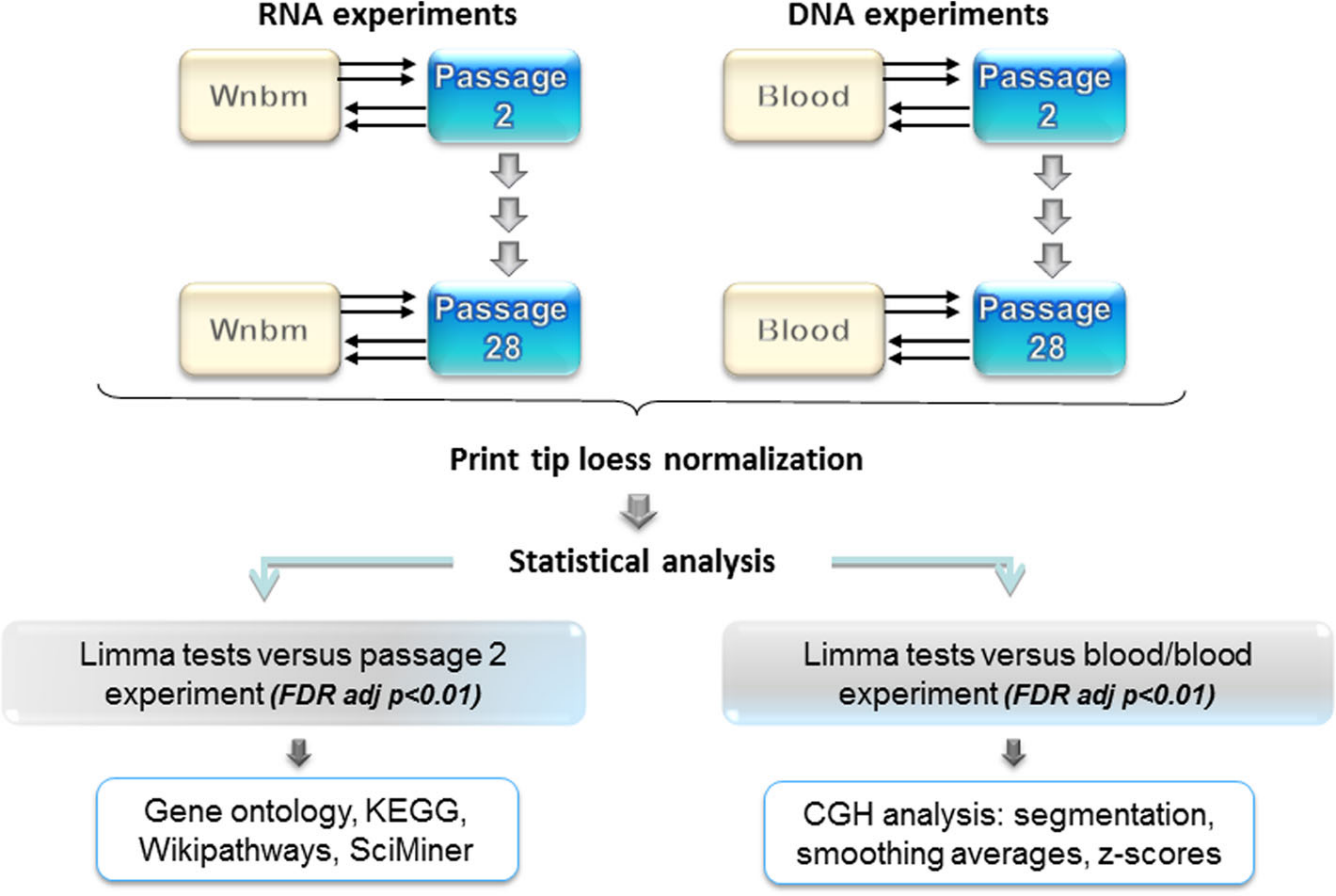
Microarray hybridization design and data analysis pipeline. Genomic DNA and total RNA profiles of MOSE cells were obtained with cDNA microarrays. Double arrows in opposite directions indicate that a common reference design plus repeated dye-swap design was used for the two series. Reference DNA was genomic DNA isolated from peripheral whole blood of adult C57BL6 male mice. Reference RNA was from a whole newborn male C57BL/6 mouse (*Wnbm*) as in previous studies [21, 22]. Test RNA and DNA samples were co-purified from the same cultures samples, labeled and hybridized on NIA-15 K cDNA microarrays as described [22]. Raw RNA and DNA datasets were separately normalized by print-tip loess with DNMAD. *Limma* (linear analysis of microarray data) analysis was performed in Pomelo2. DNA data was visualized in chromosomal format and smoothed with the WebaCGH tool [60]. Differential expression and copy number were subjected to functional genomics analyses (see details in Results section)

We have previously used the NIA-15 K microarray platform to study the transcriptomic and DNA copy number (array-CGH) profiles of established, late passage malignant, clonal MOSE cell lines [22, 23]. Figure 1 illustrates the experimental and analytical scheme followed in the present work. Distinct, common reference samples for RNA (transcriptomic) and DNA (genomic) microarray hybridizations were used. Dye-swap was applied to counteract gene-specific labeling bias [24] whereas print-tip loess normalization plus inter-slide scale percentile adjustment allowed us to minimize experimental variability of the microarray procedure arising from various sources [25]. The complete, normalized RNA dataset for 15,023 probes in 35 microarrays corresponding to 9 samples (i.e., 4 replicates in RNA samples of passages 2, 5, 10, 14, 18, 23, 25 and 28, plus 3 replicates in samples of passage 7) is available as the Additional file 1 (Urzua_RNA_dataset.xls). Passage designation is hereafter referred as pX, where X = 2, 5, 7, 10, 14, 18, 23, 25 and 28. RNA data were deposited to GEO (http://www.ncbi.nlm.nih.gov/geo/) with the accession code GSE81729.

The in vitro transformation experiment of MOSE cells [15] has been reproduced by several laboratories [17, 19, 21]. Based on multiple growth parameters, Roberts et al defined early, intermediate and late stages of MOSE transformation [19]. Early (E) stage corresponded to p4-15 while early-intermediate (E/I) comprised cells at p20-35. In the original work of Roby et al [15], p20 was the hallmark of pre-malignancy to malignancy. Consistently, E-stage cells grew as single monolayers that did not form spheroids whereas E/I-stage cells were able to invade the collagen support and formed <50 μm spheroids [19]. Therefore, as this evident phenotypic transition should rely on a gene-expression switch occurring before than p20, we aimed to examine early, pre-malignant transcriptomic and genomic changes in a shorter time-course, i.e., every 3–4 passages. Then, taking the transcriptomic profile of MOSE cells at p2 as “baseline”, paired *limma* tests with profiles of p5, 7, 10, 14, 18, 23, 25 and 28 were conducted to identify significant differentially expressed genes (DEG) during the course of the transformation process using an adjusted *p*-value < 0.01 as cut-off. The p2-baseline transcriptome assumption implies that: i) p2 cells are the closest state to in vivo, “native” transcription; and ii) any extent of differential gene expression after p2, reflects adaptation of MOSE cells to culture conditions in early passages and might reveal their intrinsic and spontaneous drift leading to aneuploidy-mediated transformation in later passages. The same rationale was applied to analyze genomic DNA (array-CGH) data for which the baseline was a self-to-self experiment conducted with a reference, normal germline DNA. In this case, we thought reasonable assuming that genomic DNA of MOSE cells at p2 would be undistinguishable from germline DNA, and that any DNA copy number variation arising in later passages would be unequivocally detected with this approach. In addition to the *limma* analysis, the whole array-CGH dataset was put in chromosomal display by applying a smoothing algorithm and z-score filters (see Methods).

**Fig. 2:**
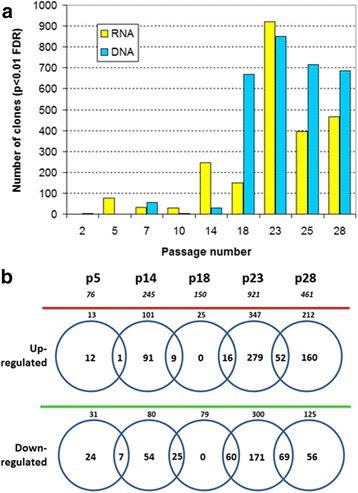
Summary of differential transcription and DNA aberrations during MOSE transformation. Chart **a** shows the number of statistically significant probes (adjusted FDR *p* < 0.01) corresponding to non-redundant cDNA clones of the NIA-15 K collection for both the RNA and the DNA experiments across MOSE culture passages as indicated. The Venn diagram in **b** depicts exclusive and common DEGs among sequential passages, each after individual comparison to passage 2

Figure 2a depicts the number of statistically significant probes (FDR adjusted *p* < 0.01) in the RNA and the DNA experiments for the MOSE cells culture passage series. Earliest DEGs detected at p5 corresponded to 13 up-regulated and 31 down-regulated genes, the latter enriched in GO:2000377 (*regulation of reactive oxygen species metabolic process*; 4 genes; *p* = 1.5e-04), GO:0006807 (*nitrogen compound metabolic process*; 16 genes; *p* = 8.6e-04) and GO:0033554 (*cellular response to stress*; 8 genes; *p* = 1.3e-03). Among genes classified under GO:2000377, *Cryab* encodes for a HSP20-related, oxidative stress protein, able to suppress nasopharyngeal tumors by interfering β-catenin function [26] while *Cyp1b1* decreases oxidative stress in endothelial cells [27]. Therefore, downregulation of both *Cryab* and *Cyp1b1* might be regarded as an oxidative stress initiator at p5. Regarding DNA results, an array-CGH data subset of about 60 clones was significantly altered at p7, but when placed in chromosomal context did not mark any loci. We cannot rule out if this DNA data actually correspond to micro-deletions and/or micro-amplifications remaining cryptic due to the limited resolution of this array platform. However, as much less significant DNA data was found in later p10 and p14, such putative cryptic gains and losses at p7 might be regarded as transient, i.e., they do not persist across successive cell sub-cultivation.

We found that transcriptional alterations were in part permanent or transitory along MOSE pre-malignant transformation. Figure 2b attempts to clarify this result by using *boolean* comparisons for selected culture passages. Only 8 out of the 44 DEG at p5 were conserved by p14. In turn, of 181 DEG at p14, 139 were transient and 34 persisted until p18. Interestingly, DNA copy number alterations dramatically emerged at p18, which was the only stage that lacked “exclusive” differential expression. In addition to the 34 genes shared with precedent p14, 76 additional genes persisted to the subsequent p23, in which cells express the tumorigenic phenotype. Thus, p18 might be considered a transitional period in which copy-number alterations emerge in the presence of a more stable and discrete transcriptional repertoire respect to earlier and later passages.

Further *boolean* comparisons were done between overlapping genes in p14 versus p18, p18 versus p23 and p23 versus p28 comparisons shown in Fig. 2b. Seventeen genes were permanently down-regulated and 3 genes were permanently up-regulated from p14 through p28. Among the first group, *Arhgef12*, *Ctsb*, *Ctsh*, *Malat1* and *Sfpq* were classified under the biological terms described in Table 1. Notably, *Arhgef12* has been described as a tumor suppressor while *Malat1* and *Sfpq* participate in biogenesis of paraspeckles (see below). The 12 remaining down-regulated transcripts included *2010111I01Rik, Sorbs2, Phf17, Ubr3* and *Fth1*, catalogued under the term GO:0046914 (*transition metal ion binding*). On the other hand, the 3 up-regulated genes persisting until p28 were *Hs6st2*, a heparan-sulfate sulfotransferase linked to EGF-like induced angiogenesis in OC cells [28], *Hmga1*, coding for a non-histone chromosomal protein recently proposed as a diagnostic urine marker in serous epithelial OC [29] and *Mmp2*, a matrix metallopeptidase implicated in OC metastasis [30]. Interestingly, though high *Malat1* expression has been linked to cell proliferation and metastasis, a recent study suggest a tumor-suppressor role of *Malat1* in gliomas through inhibition of ERK/MAPK signaling and *Mmp2*-mediated invasiveness [31], findings consistent with its down-regulation in our model.

**Table 1:** Ontology and pathway analysis of genes dysregulated at passage 14

Term^a^	Genes	Enrichment	Adj *p*-value
Up-regulated (53/101 unique genes)			
Centralspindlin complex	*Racgap1, Kif23*	72.4	2.4e-03
Chromosome passenger complex	*Cdca8, Birc5*	48.3	5.1e-03
MCM complex^b^	*Mcm2, Mcm3, Mcm6*	31.1	1.5e-03
Chromatin assembly	*Mcm2, Cenpa, Hmga1, Hmga2, Chaf1b, H2afz*	15.1	2.0e-04
Chromosome condensation	*Ncapd2, Nusap1, Top2a, Hmga2, Ccnb1*	17.3	5.0e-04
Sister chromatid segregation	*Ccnb1, Cdca8, Knstrn, Ncapd2, Nusap1, Top2a*	11.2	3.2e-04
Kinetochore	*Birc5, Ccnb1, Cenpa, Knstrn, Trp53bp1*	9.9	1.5e-02
Cytokinesis	*Racgap1, Nusap1, Kif23, Birc5, Ckap2, Anln, Prc1*	8.1	8.0e-04
Steroid metabolic process	*Lss, Hsd17b12, Fdft1, Msmo1, Ldlr, Mvd, Hmgcr*	6.1	1.3e-03
Nucleotide metabolic process	*Dhfr, Gapdh, Gmpr, Hmgcr, Pkm, Pnkp, Rrm2, Taldo1, Tk1, Tpi1*	5.8	5.2e-04
Histone binding	*Chaf1b, Hist1h4n, Mcm2, Ncapd2, Trp53bp1,Uhrf1*	5.5	2.7e-02
Spindle	*Racgap1, Kif23, Cdca8, Birc5,Dlgap5, Ccnb1, Knstrn1, Nusap1, Prc1*	5.3	1.5e-03
Extracellular matrix	*Itgb1, Gpc3, Fbln2, Fn1, Hsd17b12, Anxa2, Mmp2, Pkm, Rpsa*	4.6	3.3e-03
Mitotic nuclear division	*Anln, Birc5, Ccna2, Ccnb1, Ccnb2, Cdc20, Cdca8, Hmga2, Kif23, Knstrn, Ncapd2, Nusap1, Racgap1, Triobp*	4.3	5.0e-04
Histone deacetylase binding	*Cdc20, Hsp90ab1,Top2a*	4.2	2.4e-02
Centrosome	*Ccnb1, Kif23, Tcp1, Cdc20, Krt18, Ccnb2, Cdca8, Birc5, Dlgap5, Ckap2, Mcm3, Tacc3*	4.1	1.0e-03
Negative regulation of Wnt signaling	*Gpc3, Hmga2, Nxn*	4.0	3.4e-02
Down-regulated (35/80)			
mRNA processing^c^	*Ddx3x, Ttc14, Clk4, Rps24, Eif4a2, Sfpq, Matr3, Clk1, Rbms1, Srsf11, Zfml*	17.1	5.0e-11
Unfolded & misfolded protein binding	*Hsp90b1, Hspa5, Dnaja2, Dnajc3*	10.6	3.2e-02
Histone acetyl transferase activity	*Crebbp, Ogt*	9.1	2.1e-02
Cysteine-type peptidase activity	*Ctsb, Ctsh, Otud7b, Senp6, Usp3*	8.7	1.2e-02
Response to ER stress	*Dnajc3, Hsp90b1, Hspa5, Itpr1, Pdia3, Pdia4*	8.3	7.5e-03
Nuclear body	*Neat1, Sfpq, Malat1, Atrx, Crebbp*	7.6	3.5e-02
Regulation of chromosome organization	*Atrx, Ogt, Paxbp1, Pten, Senp6, Sfpq*	5.5	1.3e-02
Wnt signaling pathway	*Ddx3x, Ndrg2, Pten*	3.1	4.7e-02
Apoptotic signaling in response to DNA damage	*Cdip1, Nupr1*	2.8	3.3e-02
Tumor suppression^d^	*Crebbp, Ddx3x, Eef1a1, Pten, Hsp90b1, Arhgef12* *Nupr1, Pdcd4, Ndrg2*	2.9	1.3e-03

^a^Upon exclusion of repeats, unknowns and transcribed sequences, the 245 statistically significant probes at passage 14 (see Fig. 2) were reduced to 101 up-regulated and 80 down-regulated unique DEGs. These gene sub-sets were subjected to gene ontology (GO) analysis with WebGestalt (http://www.webgestalt.org/) using the hypergeometric test

^b^MCM stands for *minichromosome maintenance*

^c^Function taken from WikiPathways analysis done with WebGestalt

^d^Function derived from the TSGene database (https://bioinfo.uth.edu//TSGene1.0/). A chi-square test with Yates correction was done with GraphPad online (http://graphpad.com/quickcalcs/contingency2/)

### Dysregulated transcription prior to DNA copy number alterations

Given the particular transcriptomic and DNA copy-number profile of p18, it was reasonable to hypothesize that DEG in previous p14 could lead MOSE cells towards a genomic instability process. Table 1 shows a gene ontology (GO) analysis of the 101 up-regulated plus the 80 down-regulated transcripts at p14 in the MOSE model. Notably, among up-regulated genes, the top-3 enriched functions (>30 fold) comprised genes coding for *centralspindlin*, *chromosome passenger* and *minichromosome maintenance* (MCM) protein complexes, a set of structures essential for dynamics of mitosis. The *centralspindlin* complex is composed of the molecular motor *Kif23* and the GTPase activating protein *Racgap1*, the latter upregulated in gastric, colorectal and breast cancer [32–34]. In addition, the GO terms *chromatin assembly*, *chromosome condensation*, *sister chromatid segregation*, *kinetochore*, *cytokinesis*, *histone binding*, *spindle, mitotic nuclear division, histone deacetylase binding* and *centrosome* covered a total of 34 genes among which *Ccnb1* (Cyclin B1), *Birc5* (Survivin), *Nusap1* (NuSAP) and *Kif23* (mitotic kinesin-like protein 1) were the most recurrent. Up-regulation of genes coding for centrosome components suggest centrosome amplification in a similar fashion as experimental *PLK4* overexpression it does [35]. A recurrent observation in many solid tumors is the presence of supernumerary centrosomes proposed to have a role in aneuploidy induction and subsequent tumorigenesis [35].

Moreover, the genes *Birc5*, *Cdc20*, *Cdca8*, *Cenpa*, *Itgb1*, *Mif*, *Nusap1* and *Tacc3* have been classified under the MGI’s gene-phenotype term *abnormal mitosis* (MP:0004046; *p* = 9.0e-08). *Birc5*, a well-known anti-apoptotic gene that constitutes the *chromosomal passenger* complex and also takes part of *spindle* and *cytokinesis* terms, is an OC growth promoting factor up-regulated by the luteinizing hormone (LH) [36]. Among several hormone receptors, OSE cells express LH and FSH receptors. Gonadotropins LH and FSH increase at menopause and are considered OC predisposing factors [37]. On the other hand, the MCM complex consists of helicases needed for DNA replication that normally are bound in excess to the chromatin to counteract replicative stress. As MCM genes over-expressed transiently in MOSE cells at p14, we hypothesize that MCM depletion in later passages induce chromosomal aberrations upon overcoming a mitotic checkpoint as formerly described in HeLa cells [38]. Additional GO terms significantly enriched among up-regulated genes were *steroid metabolic process*, *nucleotide metabolic process* and *extracellular matrix*.

Regarding down-regulated genes at p14, the top enriched function was *mRNA processing* covering 11 genes (Table 1), 8 of which were correlated to *number of litters* and to *ovarian tumor frequency* in a previous transcriptomic study of our laboratory aimed to associate reproductive parameters and spontaneous tumor rates across 4 mice strains [39]. RNA processing has been increasingly connected to the DNA damage response [40], which in our results links to apoptosis through *Nupr1* and *Cdip1* (Table 1), the latter a regulator of TNF-alpha-mediated, p53-dependent apoptosis [41]. Furthermore, the splicing factor proline/glutamine-rich (*Sfpq*) has been involved in DNA double-strands break repair [42] and, together with *Neat1, Malat1, Atrx* and *Crebbp*, are catalogued under the cellular component *nuclear body* (Table 1). Interestingly, *Malat1* and *Neat1* are long non-coding transcripts that localize in a particular type of nuclear bodies termed *paraspeckles*, which consist of inter-chromatin ribonucleoprotein structures composed of *Neat1* as RNA core plus a minimal set of RNA-binding proteins including *Sfpq* [43]. Paraspeckles are emerging as key regulators of gene expression at the post-transcriptional level by its ability to sequestrate certain proteins [44] and retain mature mRNAs in the nucleus [45, 46]. In addition, *Malat1* and *Neat1* were negatively correlated to *ovarian tumor frequency*, i.e., their levels were minimal in the ovaries of mouse strains displaying the highest spontaneous tumor rates [39]. Downregulation of *Malat1* in this MOSE model along with its inverse correlation with spontaneous ovarian tumors in mice, is consistent with a recent report suggesting a tumor-suppressor role of *Malat1* in gliomas [31]. The above evidence combined suggests that MOSE cells at p14 tend to minimize the DNA damage response by downregulating RNA processing and export.

A second functional domain of downregulated genes at p14 was that comprised by *unfolded & misfolded protein binding* which overlapped with *response to ER stress*. Common to both terms were the two major ER-stress chaperones *Hspa5* alias GRP78/BiP and *Hsp90b1* alias GRP94, which provide quality control in protein folding and overall cell homeostasis [47]. Though in many cases established cancer cells display a constitutive ER-stress response to cope with proliferative demand, resist chemotherapy and evade immunity [48–50], depression of ER-stress response without evident apoptotic signaling in normal proliferating, non-malignant MOSE cells may simply indicate a decreased synthesis of secretory and membrane protein products. The third relevant down-regulated function was *tumor suppression* comprising 9 genes (Table 1), of which the phosphatase and tensin homolog (*Pten*) is the best characterized in OC. *Pten* has been implicated in papillary serous OC [51] and endometroid OC [52] whereas OC mouse models have been developed by deleting *Pten* [53, 54].

### Protein networks of genes dysregulated by passage 14

**Fig. 3:**
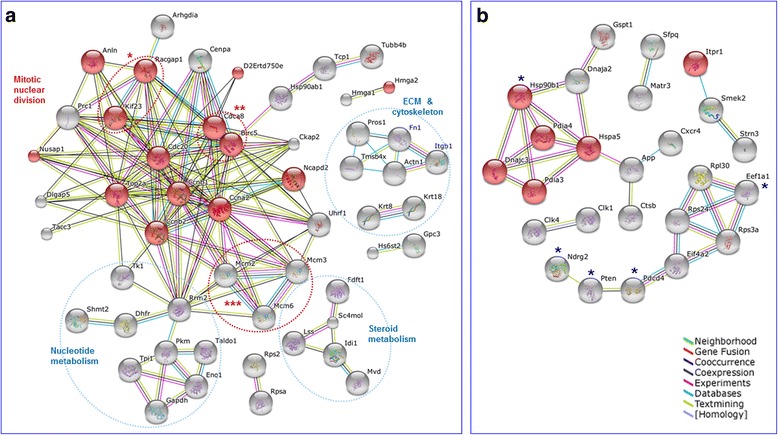
Protein-protein interactions network among genes transiently expressed at passage 14. The list of 101up-regulated (**a**) and 80 downregulated (**b**) unique gene identities were analysed with STRINGv10 (http://string-db.org/) by limiting the prediction methods to *co-expression, experiments, databases* and *textmining*. The required confidence score was set to *highest* (0.900) in (**a**) and *high* (0.700) in (**b**) and the unconnected nodes (proteins) were hidden. In (**a**), enrichment was set to the term *mitotic nuclear division* (GO:0007067; *p* = 2.7e-04) with proteins colored in red. Asterisks and red ovals depict *centralspindlin* (*), *chromosome passenger* (**) and *MCM* (***) complexes. Blue ovals enclose indicated additional GO terms; ECM stands for extracellular matrix. In (**b**), enrichment was set to *response to ER stress* (GO:0034976; *p* = 1.4e-03) and asterisks indicate tumor suppressor genes. The type of interaction is defined by color lines at bottom right. Detailed GO terms are described in Table 1. In (A), the protein *D2Ertd750e* corresponds to the updated *Knstrn* gene

Under the assumption that transcript levels are directly proportional to protein levels, we used the STRING v10 database and tool [55] to mine additional gene interrelationships derived from reported and predicted protein-protein interactions. Further functional and physical associations among genes dysregulated by p14 are shown in the networks of Fig. 3. Robust and highly interconnected networks were obtained by applying strict analysis settings that included only connected nodes (proteins), 4 of 7 possible prediction methods and highest (*0.900*)/high (*0.700*) levels of confidence scores for up-regulated/down regulated transcripts, respectively. The majority of connections were at least of 3 types, with a high frequency of experimental evidence (light purple connecting lines) supporting the network.

Major hubs in the up-regulated network (Fig. 3a) comprised 25 genes covering most of the GO terms mentioned above with *mitotic nuclear division* genes highlighted in red. Among these, the 4 hubs composing the *centralspindlin* and *chromosome passenge*r complexes plus *Ccnb1*, *Knstrn1, Nusap1*, *Dlgap5* and *Prc1*, were catalogued under the term *spindle* though the latter two are not classified as *mitotic nuclear division* genes. Similarly, genes classified under *centrosome* including *Dlgap5, Ckap2, Mcm3, Tacc3* plus 6 of the *mitotic nuclear division* genes (see Table 1) were profusely interconnected. Both the spindle and particularly the centrosome when amplified have been implicated in tumorigenesis with aneuploidy and malignant transformation [35]. Centrosome amplification might impair chromosome segregation in a cell subjected to replicative stress [56]. Consistently, as shown in Table 1, MOSE cells display upregulation of 6 genes involved in *sister chromatid segregation* including *Nusap1*, a protein that links microtubules to mitotic chromosomes and induces extensive bundling of spindle microtubules when is present in high levels [57]. Similarly, based on multiple experimental evidences, the kinetochore-related gene *Cenpa* was highly interconnected with several of the above mentioned genes. Interestingly, despite not formally catalogued as *nuclear division* related, 3 genes of the *MCM*, an hexamer protein complex needed to initiate and regulate DNA replication, showed multiple interactions with G1/S and G2/M transition-controlling cyclins *Ccnb1, Ccnb2* and *Ccna2*, as well as with cyclin inactivator *Cdc20* and the spindle assembler *Cdca8*.

Another relevant hub in network of Fig. 3a, was that of *Rrm2*, coding for the catalytic subunit of ribonucleotide reductase M2. *Rrm2* converts ribonucleotides to deoxyribonucleotides which are DNA precursors required by a cell to proliferate continuously. Coherently, *Rrm2* was in turn connected to thymidine kinase 1 (*Tk1*) and dihydrofolate reductase (*Dhfr*). *Tk1* is a proliferation-dependent enzyme involved in pyrimidine salvage pathway while *Dhfr* is essential for tetrahydrofolate recycling needed for dTMP synthesis from dUMP. *Tk1* has been considered an unspecific but useful serum cancer marker [58] and *Dhfr* was historically used as chemotherapeutic target [59]. Finally, *Rrm2* was linked both to the 3 *MCM* complex genes and to the cell division genes *Cdca8, Birc5, Cdc20* and *Top2a*.

On the other hand, the network formed among downregulated genes by p14 was much less interconnected (Fig. 3b). As expected, proteins of *response to ER stress* were highly interconnected. Of them, *Hspa5* (GRP78/Bip) connected an additional protein trio comprising the amyloid beta precursor protein (*App*), cathepsin B (*Ctsb*) and chemokine receptor 4 (*Cxcr4*). An additional hub was that of 3 ribosomal proteins plus 2 translational factors. Of interest was the presence in this network of 5 from 9 putative tumor suppressor genes (see also Table 1).

### Earliest DNA copy number changes and its cognate gene expression profile

**Fig. 4:**
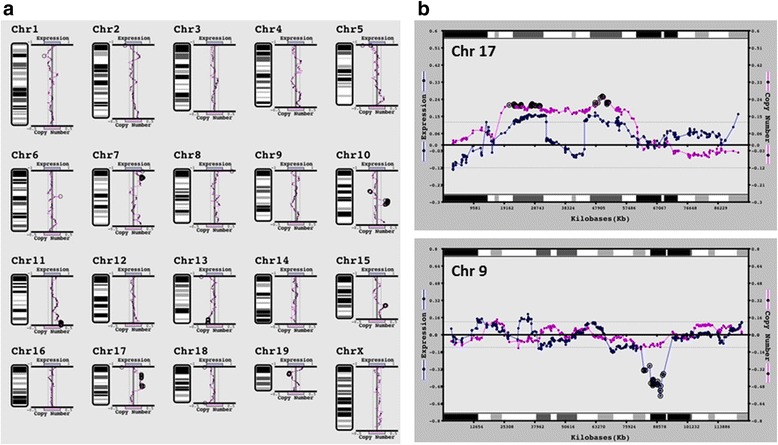
Segmental aneuploidies profile of MOSE cells prior to the malignant phenotype. Microarray-CGH data (DNA) of culture passage 18 was analyzed and visualized with the Web-aCGH tool [67]. Panel **a** shows the whole genome CGH profile. Panel **b** shows overlapped DNA copy number (purple line) and RNA transcription (blue line) for the indicated chromosomes. Smoothing window was set at 5 Mbp and *z*-score at 0.8

Figure 4a shows the DNA copy number profile of MOSE cells at p18 in an ideogram format illustrating physical chromosome positions. According to Fig. 2a, p18 is the stage when the number of DNA aberrations arises dramatically. By applying moving averages and z-score filters, discrete segmental gains were detected in chromosomes 7, 10, 11, 15 and 17 while losses were found in chromosomes 10, 13 and 19. Interestingly, in a previous genomic study we identified gains of important segments of chromosomes 10, 11 and 15 in established, late passage malignant, clonal MOSE cell lines [22]. Since the microarray-CGH approach is unable to determine complete polyploidy, we suggest that such segmental chromosome DNA aberrations detected at p18 might well correspond to the near-tetraploid genome observed by Lv et al. in this MOSE model [21]. Therefore, it is certainly possible that the apparent absence of DNA copy changes in passages earlier than p18 could correspond to a complete tetraploid genome generated by cytokinesis failure as suggested by these authors [21]. Note that 7 genes catalogued under *cytokinesis* and two of the 4 genes composing the *chromosome passenger complex*, a key structure engaged in the orderly exit from mitosis [60], are overexpressed by passage 14 (Table 1). As mentioned above, *Birc5* (Survivin) expression promotes OC growth via LH stimulation [36] and has been found overexpressed in many cancers [61]. Besides its anti-apoptotic role, overexpression of survivin decreases nucleation of centrosomal microtubules affecting mitotic spindle dynamics and cytokinesis [62].

As shown in Fig. 4b, transcriptional data was superimposed over array-CGH data. A large 41 Mbp segment of chromosome 17 was gained and, upon data smoothing, showed a concomitant trend of increased expression in two adjacent segments. This chromosomal segment contained nearly 200 genes for which proteolysis, cell-cycle, ribosome and RNA processing were the predominant functional terms (data not shown). In contrast, an 8.2 Mb segment of chromosome 9 showed no copy number alteration but apparent downregulation of 15 neighbor genes. Later by passage 23, DNA copy number changes persisted with those of chromosomes 11 and 17 enlarged in size while transcriptional changes dramatically increased (data not shown). Over 500 genes were differentially expressed and enriched in focal adhesion (19 genes, R = 10.8, *adjP* = 6e-13), TNF-alpha NF-kB signaling pathway (15 genes, R = 8.7, *adjP* = 2.8e-9), TGF-beta receptor signaling pathway (8 genes, R = 5.7, *adjP* = 1e-4), ECM-receptor interaction (8 genes, R = 11.2, *adjP* = 6.9e-6), cell cycle (9 genes, R = 7.6, *adjP* = 2.4e-5) and ribosomal proteins (33 genes, R = 31, *adjP* = 1e-15). The oncogenes and oncogene-related transcripts *Myc, Mycbp, Ndrg1, Akt1, Fgfr1op2, Hras1, Rap1b, Rhoa, Rsu, Lyn, Rala* and *Tet3* were also within this 500-subset. Overall, the functional profile of cells at p23 is consistent with a malignant phenotype, in which a few gene expression modules were identifiable in the chromosomal context although not physically related to DNA dosage variation in a large extent. These results suggest that aneuploidy in this MOSE model emerges with genomic aberrations at p18 in which chromosomal-driven transcription is not dominant but apparently follows a more complex pattern that drives to tumorigenesis.

### Mining passage-14 dysregulated genes in clinical phenotypes of human tumors

**Fig. 5:**
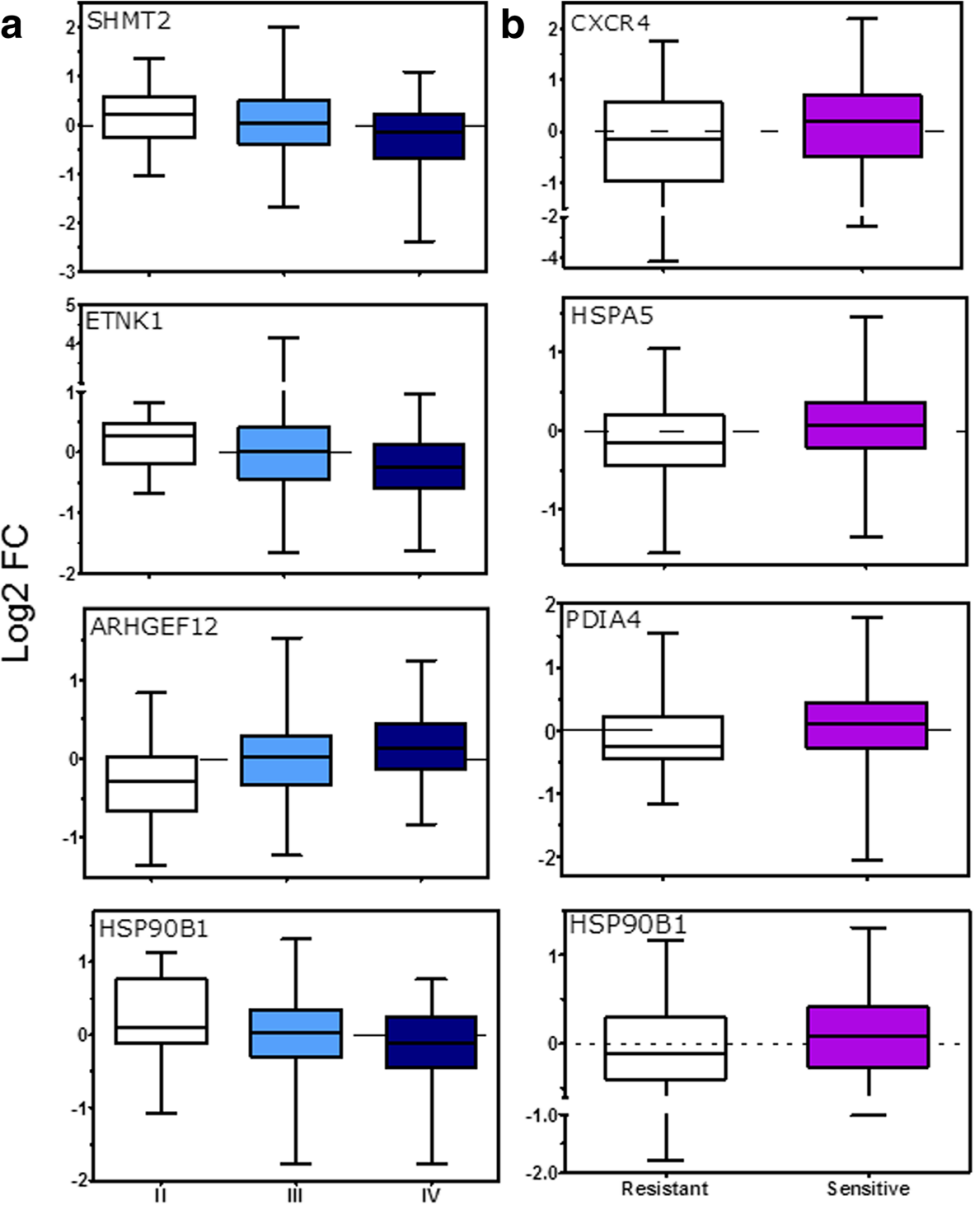
Human ovarian tumor analysis of MOSE genes dysregulated at passage 14. Gene transcription data for human OC tumors as well clinical data was freely available from the TCGA project website. Mouse gene symbols were converted to their corresponding human orthologs. For the *tumor stage* data mining (**a**), data for 473 patients was used. For the platinum status (**b**), data for 289 patients was available. *Limma* tests were applied as described in Methods. Selected genes shown were *adj p* < 0.05 for *tumor stage* (**a**) and raw *p* < 0.005 for *platinum status* (**b**)

The Cancer Genome Atlas (TCGA) is an open repository of molecular and clinical information on various tumors including OC. The TCGA ovarian expression dataset contains log2 data on 11,864 coding genes for 489 cases of high-grade ovarian serous adenocarcinomas [63]. Available clinical data includes tumor stage, grade and residual disease, primary therapy outcome, progression, and platinum response among others. To gain insight into the possible relevance of premalignant gene dysregulation in the MOSE model related to human ovarian tumors, the expression levels the 181 DEG by p14 were mined in the ovarian TCGA dataset. *Limma* ANOVA and t-tests were performed among ovarian tumor profiles according to *platinum status* (PS) *progression-free status* (PFS) and *tumor stage* (TS). For the last case, sub-stages A, B and C were collapsed into single II, III and IV stage categories. Statistical results were ranked and genes with an adjusted *p*-value < 0.05 and raw p-value <0,005 were selected. On these lists, the 181 DEG list in p14 MOSE cells was filtered out. PS resulted in 8 genes, PFS in 3 genes and TS in 6 genes present in the mouse 181 DEG at p14. SHMT2 and ARHGEF12 were the top significant genes. All genes except SHMT2 and ETNK1, corresponded to down-regulated genes in MOSE cells at p14. Figure 5 shows these two plus other 5 significant genes. Despite HSP90B1 (GRP94) expression has been linked to cancer growth and metastasis [64], decreased HSP90B1 expression as tumor progresses is consistent with its proposed role as tumor suppressor in the TSGene database [65]. The ARHGEF12 pattern was paradoxically opposite in view of its proposed role as tumor suppressor in breast and colorectal cancer [66]. On the other hand, the *platinum status* pattern of all genes in Fig. 5b, including HSP90B1, suggest that their downregulation would indicate a poor chemotherapeutic response to cisplatin, the first line agent to treat OC.

## Conclusion

The OSE has been regarded as a possible origin of ovarian cancer caused by a chronic damage-repair cycling upon repetitive ovulatory events. Such damage can be experimentally replicated in vitro leading to spontaneous cell transformation. The present study was an attempt to decode the early, pre-malignant genomic and transcriptomic changes occurring prior to the development of malignant capacity. Initial segmental aneuploidies were detected at passage 18 in discrete segments of chromosomes 7, 10, 11 and 17. These early DNA aberrations were preceded by transient differential expression of 181 genes at p14. Based on previous cytogenetic analysis of this model by Lv et al [21], the functional profile of the 101 up-regulated genes at p14 enabled us to suggest that an abnormal mitotic process is taking place at p14 and might include centrosome amplification, cytokinesis failure and replicative stress. On the contrary, down-regulated genes indicate impaired RNA processing, response to ER stress and tumor suppression. The time course of transcriptional and genomic alterations of MOSE cells identified in the present study, particularly those between p14 and p18, further agree with findings of Lv et al [21]. Specifically, we propose that profile of MOSE cells at p14 would correspond to tetraploid cells leading to a near-tetraploid stage at p18, in which some of the segmental chromosome aberrations could initiate and perpetuate the tumorigenic drive. The gene expression profile of later passages indicates the rise of a frankly malignant phenotype. Among the limitations of this study is the difficulty to extrapolate in vitro genomic and transcriptional changes to a pre-neoplasia arising *in situ* at the OSE. It has been described that inclusion cysts present in aged ovaries may experience a particular epithelial-mesenchymal transition harboring pre-metaplasic characteristics. The interaction of OSE with diverse cell types including interstitial and stromal ovarian cells as well as the immune cells infiltrating the senescent ovary, will certainly modulate the fate of a pre-neoplastic tetraploid cell formed as a consequence of repeated ovulation.

## Methods

### Cell cultures and nucleic acids extraction

Mouse ovarian surface epithelial (MOSE) cells from 8-weeks old female C57BL6 mice were isolated and cultured as described [15]. Total RNA and genomic DNA from culture passages 2, 5, 7, 10, 14, 18, 23, 25 and 28 were obtained with TRizol reagent following the standard protocol. The in vitro transformation experiment of MOSE cells has been described by Roby et al [15].

### DNA microarray experiments

Conditions of RNA and DNA labeling, hybridization, washes and scanning were performed as previously described [22, 23]. Microarrays were used in test versus reference (2-channel) format. The overall experimental scheme is shown in Fig. 1, top section. Gene transcription (RNA) and genome (DNA) microarray hybridizations were performed using repeated dye-swap with common reference for both the RNA and the DNA series. RNA from a C57BL6 newborn male mouse (Wnbm) and DNA from C57BL6 male peripheral blood were the reference samples for the two series of experiments, respectively.

### Microarray data analysis

Slide images were extracted with the GenePix Pro 5.0 software. GPR files were deposited at the NCI’s microarray database, mAdb (https://madb.nci.nih.gov/), and normalized with loess, background “half” correction and multi-slide scale adjustment using the DNMAD tool. Of the total 15,260 cDNA clones in this platform, 11,218 were annotated as known transcripts including 1,025 transcribed loci, 200 of which are moderately or strongly similar to known genes. For the array-CGH experiments shown here, 12,275 probes were physically mapped to the current build of the M musculus genome resulting in a mean coverage of 221 Kbp/probe.

### Statistical and bioinformatic analysis

Normalized log2 ratio RNA and DNA data consisted of 4 replicates per sample passage with a total of 71 microarray experiments. *Limma* t-tests with FDR control were done at Pomelo II (http://pomelo2.iib.uam.es/). For RNA data, each of all passages from p4 to p28 was compared against p2 data, regarded as baseline. DEG were cut-off with adj *p* < 0.01 and subjected to GO, KEGG and WikiPathway analysis at WebGestalt (http://www.webgestalt.org/) and VLAD http://proto.informatics.jax.org/prototypes/vlad/. Network analysis was done with STRING v10 (http://string-db.org/). Normalized DNA results were compared against a reference-to-reference experiment. Data was displayed in chromosomal context and smoothed with WebaCGH [67]. TCGA data was at https://tcga-data.nci.nih.gov/docs/publications/ov_2011/ and analyzed with Pomelo II.

## Additional file

Additional file 1: Urzua_RNA_dataset. Contains the complete, loess normalized dataset of log2 ratio data values for 15,023 cDNA probes in 35 microarrays. Column A contains clone (gene) identity and gene description. (XLS 10723 kb)
